# 

**Published:** 1979

**Authors:** 


### Contents

3 Postgraduate Medical Education in the South West
J. E. Cates

13 Obituary
Dr. R. I. S. Gordon, M.D., F.R.C.P., F.R.C.R.

15 Abstracts

